# What Is Clinical Anatomy?—A Consensus Statement From the American Association of Clinical Anatomists

**DOI:** 10.1002/ca.70040

**Published:** 2025-10-21

**Authors:** Joe Iwanaga, Kathleen Bubb, Mathangi Rajaram‐Gilkes, Geoffroy Noel, Arada Chaiyamoon, David Ezra, Ameed Raoof, Guenevere Rae, Estomih P. Mtui, Alan J. Detton, Mahindra Kumar Anand, Kazzara Raeburn, Mi‐Sun Hur, Hee‐Jin Kim, Laligam N. Sekhar, Yoko Tabira, Koichi Watanabe, Mohammed K. Khalil, Anthony D' Antoni, Marios Loukas, Robert J. Spinner, Philip J. Adds, R. Shane Tubbs

**Affiliations:** ^1^ Department of Neurosurgery, Clinical Neuroscience Research Center Tulane University School of Medicine New Orleans Louisiana USA; ^2^ Department of Neurology, Clinical Neuroscience Research Center Tulane University School of Medicine New Orleans Louisiana USA; ^3^ Department of Structural & Cellular Biology Tulane University School of Medicine New Orleans Louisiana USA; ^4^ Department of Neurosurgery and Ochsner Neuroscience Institute Ochsner Health System New Orleans Louisiana USA; ^5^ Dental and Oral Medical Center Kurume University School of Medicine Fukuoka Japan; ^6^ Division of Gross and Clinical Anatomy, Department of Anatomy Kurume University School of Medicine Fukuoka Japan; ^7^ Division of Anatomy, Department of Radiology Weill Cornell Medicine New York USA; ^8^ Anatomical Sciences, Department of Medical Education Geisinger Commonwealth School of Medicine Scranton Pennsylvania USA; ^9^ Anatomy Division, Department of Surgery University of California San Diego San Diego California USA; ^10^ Department of Anatomy, Faculty of Medicine Khon Kaen University Khon Kaen Thailand; ^11^ School of Nursing Sciences Academic College Tel Aviv Jaffo Israel; ^12^ Department of Anatomy and Anthropology, Sackler Faculty of Medicine Tel‐Aviv University Tel‐Aviv Israel; ^13^ Department of Physical Anthropology Natural History Museum Cleveland Ohio USA; ^14^ Faculty of Biomedical Sciences at Kaiser Permanente School of Medicine Pasadena California USA; ^15^ Department of Pathology & Cell Biology Columbia University Vagelos College of Physicians and Surgeons New York New York USA; ^16^ Department of Anatomy Rama Medical College & Research Centre Hapur India; ^17^ Department of Anatomical Sciences St. George's University Grenada; ^18^ Department of Anatomy Daegu Catholic University School of Medicine Daegu South Korea; ^19^ Division in Anatomy and Developmental Biology, Department of Oral Biology Human Identification Research Institute, BK21 Four Project Yonsei University College of Dentistry Seoul Korea; ^20^ Department of Neurological Surgery UW Medicine Seattle Washington USA; ^21^ Department of Biomedical Sciences University of South Carolina, School of Medicine Greenville Greenville South Carolina USA; ^22^ Department of Medical Education and Scholarship Rowan‐Virtua School of Osteopathic Medicine Stratford New Jersey USA; ^23^ Department of Clinical Anatomy Mayo Clinic Rochester Minnesota USA; ^24^ Department of Pathology St. George's University, School of Medicine West Indies; ^25^ Nicolaus Copernicus Superior School, College of Medical Sciences Olsztyn Poland; ^26^ Department of Neurosurgery Mayo Clinic Rochester Minnesota USA; ^27^ Department of Anatomy, St George's University of London London UK; ^28^ Department of Surgery Tulane University School of Medicine New Orleans Louisiana USA; ^29^ University of Queensland Brisbane Australia

**Keywords:** anatomy, clinical anatomist, dentistry, education, gross anatomy, head and neck, international, medicine, research, surgery

## Abstract

At the 42nd Annual Meeting of the American Association of Clinical Anatomists (AACA) in Bellevue, Washington, June 2025, two inaugural events—the Clinical Anatomy Fireside Chat (CAFC) and the Clinical Anatomy Symposium: Head and Neck 2025 (CAS)—fostered rich dialogue on the evolving role and operational definition of clinical anatomy. Experts from various clinical and anatomical disciplines explored the meaning of clinical anatomy, highlighting the absence of a universal definition despite its frequent use in education and research. Through these interdisciplinary discussions, a consensus emerged: clinical anatomy is not defined solely by the possession of clinical credentials but by the integration of anatomical knowledge and clinical relevance, achieved most effectively through collaboration. Clinical anatomy education and research require different depths of clinical knowledge depending on the audience and objective, and meaningful collaboration can bridge gaps in expertise. The symposium further illustrated that high‐quality clinical anatomy emerges from mutual respect and reciprocal insight between clinicians and anatomists. This article presents a consensus statement developed by AACA representatives and invited speakers, affirming that collaboration is not only foundational to the practice of clinical anatomy but also fundamental to its definition. These conclusions aim to guide future educational models, research strategies, and interdisciplinary partnerships in the field of clinical anatomy.

## 1 Introduction

In June 2025, during the 42nd Annual Meeting of the American Association of Clinical Anatomists (AACA) in Bellevue, Washington, two inaugural events were introduced: the Clinical Anatomy Fireside Chat (CAFC) and the Clinical Anatomy Symposium: Head and Neck 2025 (CAS). The CAFC session focused on exploring the concept of clinical anatomy through open dialogue among experts, while the CAS session brought that concept to life through a series of presentations that showcased clinical anatomy across various specialties. These events brought together invited speakers from both within and outside the AACA from 10 different countries, including clinicians and anatomists representing diverse clinical and academic disciplines. Together, they engaged in interdisciplinary discussions spanning clinical practice, anatomical research, and education.

The purpose of this article is to summarize the central themes discussed during these sessions, to articulate insights that emerged in their aftermath, and to address key conceptual questions that remained unresolved. This paper reflects a collaboratively developed perspective—a preliminary consensus shaped by AACA representatives and session speakers—on the evolving definition and role of clinical anatomy.

Although a formal consensus statement was not originally planned during the meeting, the topic was extensively discussed. A draft was later developed collaboratively and circulated via email, through which consensus was reached by majority agreement among contributing authors.

By sharing these reflections, we aim to foster continued dialogue, promote meaningful collaboration between clinicians and anatomists, and support the advancement of clinical anatomy as an inclusive and integrative discipline.

## 2 What Is Clinical Anatomy?

At one point, a fundamental question was raised: “How do you define clinical anatomy?” Although the term “clinical anatomy” is widely used, each speaker offered a different interpretation. This variation stems from the fact that, despite numerous descriptions in the literature, no universally accepted definition exists.

The roots of the AACA trace back to February 18, 1983, when 18 surgeons and anatomists gathered to establish a forum for more effective dialogue between those who practice the application of anatomy in a clinical setting and those who teach and research anatomy (American Association of Clinical Anatomists 2025). The mission of the AACA is “the advancement of clinical anatomy knowledge and anatomical services through education, research, and scholarship.”

So, again, what exactly is clinical anatomy?

One hint at the long‐term struggle with defining this term is to examine earlier anatomical textbooks that have attempted to use various terms to capture the essence of this discipline. For example, “applied anatomy” was used by von Bardeleben, Haeckel, Frohse, and Ziehen in their text *Atlas of Applied (Topographical) Human Anatomy* (von Bardeleben et al. 1906). Interestingly, the subtitle of *Gray's Anatomy* has varied over the years. For example, the first edition was *Anatomy: Descriptive and Surgical*. Over the years, it evolved into *Gray's Anatomy: Descriptive and Applied* (e.g., 34th edition) and *Gray's Anatomy: The Anatomical Basis of Medicine and Surgery* (e.g., 38th edition), and it is now known as *Gray's Anatomy: The Anatomical Basis of Clinical Practice* (e.g., 42nd edition).

“Practical” has been used in the title of many older anatomical texts as well. Trying to encapsulate this topic, Ranney (1882), in his textbook *Practical Medical Anatomy*, said, “Anatomy is not alone a mere preparatory drill to the practical branches, but a source of every varying utility in all the walks of professional life.” Eckley and Eckley (1899) furthered this train of thought when they wrote, “the growing tendency to specialize in the practice of medicine has reacted on the branch of medical science that continges every medical specialty–anatomy; with the result that our schools are compelled to keep pace in teaching this branch.”

In 1988, Dr. Ralph Ger (Ger and Scothorne 1988) launched the journal *Clinical Anatomy* (the official journal of both AACA and the British Association of Clinical Anatomists, BACA) with an inaugural Editorial entitled “What is clinical anatomy? Does it need, or deserve, a new journal?” In this piece, Ger emphasized that the journal was “dedicated to the dissemination of new knowledge of all aspects of anatomy of relevance to clinical science and practice.” Three decades later, Tubbs (2017) revisited this foundational question in an Editorial, encouraging readers to reflect on Ger's original statement and its continued relevance. More recently, Zhang et al. (2023) and several other authors have described clinical anatomy as “a subject that merges clinical diagnosis and treatment into anatomy learning.” Nevertheless, the term continues to be used anecdotally or empirically in many contexts (D'Antoni et al. 2019; Clifton et al. 2020).

Similarly, “Surgical Anatomy” has been used in the past and current textbooks. Campbell (1922) wrote, “The purpose of surgical anatomy is to present anatomic facts in terms of their clinical values, and thus properly appraise those structures and regions which have a practical interest for the surgeon.” However, even detailed, multi‐volume textbooks on surgical anatomy have struggled to define this field. For example, (Skandalakis' Surgical Anatomy 2004), in his preface, commented on the impetus of the book,

My hope is that we have articulated our collection of anatomic pearls to form a precious possession on paper for the student, the resident, and the practicing surgeon.

Moore et al. (2014) provided one of the most frequently cited textbook definitions:

Clinical anatomy emphasizes aspects of the structure and function of the body important in the practice of medicine, dentistry, and the allied health sciences. It encompasses both the regional and systemic approaches to studying anatomy and stresses clinical application.

If we follow Moore et al.'s logic, clinical anatomy may be best viewed as a discipline, parallel to, or a specialized branch within “gross anatomy.” Therefore, clinical anatomy can be characterized as anatomy for patient care.

## 3 Clinical Anatomy Education

The term *clinical anatomy* is commonly used in medical education, particularly in reference to the integration of basic sciences, such as anatomy, with clinical applications (Khalil et al. 2021; D'Antoni et al. 2019). It has also been described as the merging of operative surgery with gross anatomy (Kagan 2002). Accordingly, clinical anatomy education can be understood as instruction rooted in the discipline of clinical anatomy. More recently, *evidence‐based anatomy* (EBA) has emerged as an important approach for applying anatomical knowledge to clinical practice (Yammine 2014; D'Antoni et al. 2022, 2025).

However, effective clinical anatomy education demands some level of clinical knowledge. Standard clinical anatomy textbooks typically offer only limited general clinical content. As specialization increases, so does the need for field‐specific anatomical and clinical knowledge. For instance, skull base surgery requires familiarity with highly specialized anatomical regions—such as the facial recess for the mastoidectomy, retaining ligaments related to the face, or the paramedian triangle of the cavernous sinus—that are rarely covered in general anatomical texts (Drazin et al. 2017; Watanabe et al. 2023; Komune et al. Early View).

Therefore, the depth and scope of clinical anatomy education depend on who the learners are:

- For clinical students, foundational clinical knowledge may suffice.
- For fully trained clinicians in specialized fields, advanced and highly specific anatomical and clinical knowledge is essential. This type of clinical anatomy education can be challenging for anatomists without a thorough clinical training in the same field, especially when working alone.

## 4 Clinical Anatomy Research

Clinical anatomy research also builds upon the clinical anatomy discipline, but unlike education, its goal is to generate new knowledge. Producing novel findings often requires choosing niche topics, which in turn demands a detailed understanding of the clinical field. This level of insight is difficult to attain without years of direct clinical experience. For example, Ciporen et al. (2010) conducted a cadaveric study to evaluate whether a multiportal endoscopic approach to the central skull base improves surgical access and maneuverability in the central anterior cranial fossa. So, does this mean that only specialists can conduct meaningful clinical anatomy research? Not necessarily.

During the CAFC, one speaker stated, “The key to clinical anatomy is collaboration.” When an anatomist, with expertise in anatomical methodology and research design, partners with a clinical specialist, the collaboration can lead to meaningful and innovative work in clinical anatomy. For instance, the petroclival ligament (PCL), also known as the petroclinoid ligament, is a surgically significant structure that is rarely addressed in standard medical education (Iwanaga, Altafulla, et al. 2020). It is essential to acknowledge that clinical anatomy research developed from a single perspective—or “one eye”—can be susceptible to bias. The PCL has traditionally been considered a distinct ligament within the skull base because it is consistently encountered during surgery. However, anatomical and histological analyses have demonstrated that the PCL is merely a thickening of the dura mater, as some researchers had previously suggested anecdotally (Iwanaga et al. Forthcoming). This highlights the importance of integrating both anatomical and clinical expertise to deepen our understanding of such structures.

Misunderstandings between clinicians and anatomists often arise from a lack of collaboration. For instance, while the ligamenta flava are anatomically composed of a single layer, many spine surgeons believe they consist of two. Following interdisciplinary dialogue, anatomists and spine surgeons conducted a comprehensive gross anatomical and histological investigation and found that the structure consists of a single layer. Without open dialogue and collaborative investigation, such discrepancies might have remained unrecognized (Iwanaga, Ishak, et al. 2020).

## 5 Can Anatomists Without Clinical Licenses Practice Clinical Anatomy?

At the end of the CAFC, another question was raised:

Can someone without a clinical license practice or contribute to clinical anatomy?

Opinions may differ, but within the AACA, the prevailing belief is:

Yes. Both clinical anatomy education and research can be conducted by anatomists without clinical licenses or training, provided they engage in genuine and meaningful collaboration with clinical specialists. This collaboration should go beyond simply exchanging opinions or sharing cadaveric donors. True integration requires both parties to actively engage in discussion, combining their knowledge and experience. Through this process, anatomists can gain insight into the clinical perspective, while clinicians can deepen their understanding of anatomical principles.

## 6 Clinical Anatomy Symposium

At the conclusion of the annual meeting, the *Clinical Anatomy Symposium* Head and Neck 2025 featured speakers from a range of specialties, including skull base surgery, diagnostic imaging, and dental, oral, and maxillofacial surgery. What was observed was striking: *every presentation showcased highly focused clinical anatomy research and education, each rooted in interdisciplinary collaboration*. While many clinical anatomists likely already understood the importance of collaboration in producing high‐quality clinical anatomy, *this symposium provided* clinical anatomists *with the opportunity to finally articulate their understanding*.

The clinical anatomy community stands at the threshold of a new era—one where clinical anatomy research and education are no longer siloed disciplines but are shared endeavors shaped by genuinely sustained collaborations. With mutual respect and the blending of clinical insight and anatomical precision, we can envision a future in which our collective efforts redefine understanding, elevate education, and ultimately improve patient care.

The authors of this consensus paper (made up of 17 clinically trained and 6 anatomically trained professionals) define clinical anatomy as follows:

Clinical anatomy is an integrative discipline that applies anatomical knowledge to clinical practice, research, and education, with the ultimate goal of improving patient care. It is not defined by the possession of clinical credentials, but rather by the deliberate and informed integration of anatomical expertise with clinical context and relevance. Such collaboration fosters meaningful and innovative advancements in the field of clinical anatomy.

## 7 Conclusions

Clinical anatomy is a discipline defined not by credentials, but by collaboration—among those who teach, study, and apply anatomy in clinically meaningful ways. As emphasized during the AACA 2025 annual meeting, this collaboration must actively include clinicians, without whom the role of clinical anatomy risks being confined solely to student education. At the same time, rapid advances in medical technology and the rise of noninvasive, “borderless” (interdisciplinary and international) practices are transforming the clinical landscape. These shifts demand an equally adaptive anatomical foundation. To meet this moment, clinicians from all fields must come together across traditional boundaries, and clinical anatomists must continue to serve as integrative leaders. *Based on the principles outlined in this consensus, it is evident that the AACA and other clinically oriented anatomical organizations must actively promote and facilitate greater engagement and collaboration with the clinical community moving forward*. This shared commitment is not just timely—it is essential for shaping the future of medicine and anatomy alike.

## Disclosure

This article was authored by the executive committee and council members of the AACA, and the speakers of the CAFC and CAS.

## Data Availability

Data sharing not applicable to this article as no datasets were generated or analysed during the current study.
